# Novel ophthalmic formulation of myriocin: implications in retinitis pigmentosa

**DOI:** 10.1080/10717544.2019.1574936

**Published:** 2019-03-11

**Authors:** Chiara Bianca Maria Platania, Michele Dei Cas, Simona Cianciolo, Annamaria Fidilio, Francesca Lazzara, Rita Paroni, Rosario Pignatello, Enrica Strettoi, Riccardo Ghidoni, Filippo Drago, Claudio Bucolo

**Affiliations:** a Department of Biomedical and Biotechnological Sciences, School of Medicine, University of Catania, Catania, Italy;; b Department of Health Sciences, University of Milano, Milano, Italy;; c Drug Sciences Department, University of Catania, Catania, Italy;; d NANO-i – Research Center on Ocular Nanotechnology University of Catania, Catania, Italy;; e CNR Neuroscience Institute, Pisa, Italy;; f Center for Research in Ocular Pharmacology-CERFO, University of Catania, Catania, Italy

**Keywords:** Nanostructured lipid carriers, ocular drug delivery, myriocin, retinitis pigmentosa

## Abstract

Myriocin is an antibiotic derived from *Mycelia sterilia*, and is a potent inhibitor of serine palmitoyltransferase, the enzyme involved in the first step of sphingosine synthesis. Myriocin, inhibiting ceramide synthesis, has a great potential for treatment of diseases characterized by high ceramide levels in affected tissues, such as retinitis pigmentosa (RP). Drug delivery to the retina is a challenging task, which is generally by-passed through intravitreal injection, that represents a risky invasive procedure. We, therefore, developed and characterized an ophthalmic topical nanotechnological formulation based on a nanostructured lipid carrier (NLC) and containing myriocin. The ocular distribution of myriocin in the back of the eye was assessed both in rabbits and mice using LC-MS/MS. Moreover, rabbit retinal sphingolipid and ceramides levels, after myriocin-NLC (Myr-NLC) eye drops treatment, were assessed. The results demonstrated that Myr-NLC formulation is well tolerated and provided effective levels of myriocin in the back of the eye both in rabbits and mice. We found that Myr-NLC eye drops treatment was able to significantly decrease retinal sphingolipid levels. In conclusion, these data suggest that the Myr-NLC ophthalmic formulation is suitable for pharmaceutical development and warrants further clinical evaluation of this eye drops for the treatment of RP.

## Introduction

Myriocin, a natural compound with immunosuppressive activity, worked as a template compound for the development of fingolimod, a drug approved for the treatment of multiple sclerosis. Fingolimod retained the immunosuppressant activity of myriocin but not the inhibitory activity on serine palmitoyltransferase (Zécri, 2016), the enzyme involved in the first step of sphingolipids (i.e. ceramides) synthesis. The dysregulation of ceramide biosynthesis in retinitis pigmentosa (RP), a genetically heterogeneous disorder causing photoreceptor degeneration and blindness, was hypothesized for the first time in 2004. The mutation of ceramide kinase like gene (*CERKL*) was associated with autosomal recessive RP (RP26) (Tuson et al., 2004). The endogenous substrate of *CERKL* has not been identified yet. CERKL lipid kinase activity has not been confirmed as well, but the role of *CERKL* in the regulation of the retinal sphingolipid metabolism was proven by Garanto et al. (2013). The sphingolipid metabolism was found to be altered in the retinal degeneration 10 (rd10) mouse model of RP (Strettoi et al., 2010). Particularly, the levels of pro-apoptotic ceramide were found to be higher in the retina of rd10 mice compared to control mice.

It has been demonstrated that topical ocular administration of myriocin preserves photoreceptor structure and function in a mouse model of retinitis pigmentosa (Strettoi et al., 2010), assessed by electroretinography (ERG) as well as by morphological and biochemical tests (Piano et al., 2013). Moreover, the rationale for using ceramide-synthesis inhibitors to reduce photoreceptor apoptosis in retinitis pigmentosa has also been reviewed (Guadagni et al., 2015). Myriocin nanotechnological eye drops formulations, myriocin-loaded solid lipid nanoparticles (Myr-SLN), were also tested in animal model of RP, along with myriocin intravitreal injection in rd10 mice (Strettoi et al., 2010; Piano et al., 2013).

Nanotechnological drug delivery systems are characterized by several advantages, such as noninvasive administration, biocompatibility, biodegradability, bio-adhesive properties, high encapsulation efficiency and loading capacity, good bioavailability for poorly soluble drugs, good targeting and controlled release (Ameeduzzafar et al., 2018; Imam et al., 2018; Baig et al., 2016). These characteristics led to the fruitful exploitation of nanotechnology for ocular drug delivery. Nanostructured lipid carrier (NLC) formulations, a second generation of lipid nanoparticles, have shown better characteristics compared to SLN (Battaglia and Gallarate, 2012; Araujo et al., 2011; Puglia et al., 2015; Puglia et al., 2018). In fact, SLN are characterized by some drawbacks, such as drug expulsion during storage (Battaglia et al., 2016) and lower drug loading, compared to NLC (Müller et al., 2002). The NLC are composed by blends of lipids with different melting temperatures, but solid at body temperature. Therefore, during the cooling of the lipid mixture, the liquid (oily) fraction causes nano-structural imperfections (nano-compartments of oil) in lipid nanoparticles. These oil compartments in the solid matrix increase the drug solubility, thus augmenting the total drug loading capacity of NLC, compared to SLN (Puglia et al., 2018; Müller et al., 2002). The aim of the present study was the preparation and characterization of a novel ophthalmic formulation based on NLC able to deliver myriocin to the back of the eye after topical administration. Furthermore, we assessed retinal sphingolipid levels after Myr-NLC eye drops treatment.

## Materials and methods

### Materials

Myriocin (Myr) from *Mycelia sterilia* ≥ 98% (HPLC) powder was purchased from Sigma-Aldrich (Milan, Italy); 14-hydroxy-myriocin was prepared in our laboratory by chemical reduction of the carbonyl group, purified and characterized by mass spectrometry. Sphingolipids standards were purchased by Avanti Polar Lipids (Alabaster, USA). Gelucire® 44/14 (lauroyl polyoxyl-32 glycerides) was donated by Gattefossè (France). Mygliol 812® (triglyceride capric/caprylic acids) was purchased from Farmalabor (Italy). Tween 80 and benzalkonium chloride were purchased from Sigma-Aldrich (Milan, Italy).

Methanol (MeOH), acetonitrile, ammonium formate, acetic acid, potassium hydroxide (KOH) and formic acid (all of analytical grade) were supplied by Merck (Darmstadt, Germany). Water was MilliQ grade. 

## Methods

### Preparation of blank NLC

Unloaded NLC (NLC0) was prepared using an adapted melt-emulsification and ultrasonication technique (Silva et al., 2011). Firstly, the aqueous phase was prepared at room temperature adding to distilled water the surfactant Tween 80 (2.5% w/v) and the preservative benzalkonium chloride of (0.05% w/v), then the aqueous phase was stored at 4 °C. The lipid matrix was prepared by heating Gelucire 44/14 (10% w/v) and Mygliol 812 (5% w/v) to 75 °C (according to our pre-formulation studies, data not shown). The aqueous phase, heated to the same temperature of the lipid matrix, was slowly added to the melted lipid matrix. Then the mixture was continuously stirred at 500 rpm. A pre-emulsion was thus obtained, cooled and then sonicated using a probe sonicator (S-250A analog Sonifier; Branson), set at 70% amplitude for 15 min, in order to form a nano-emulsion. After sonication, the NLC formulations were stored at 4 °C. The characterization analysis of the NLCs was carried out 24 h after preparation, in order to reach system equilibration.

### Preparation of myriocin-loaded NLCs

Myriocin loaded NLC (Myr-NLC) were prepared with the same technique of unloaded NLC. Myriocin (1 or 2 mg/ml, respectively for batches NLC1 and NLC2) was added to the lipid matrix, heated at 75 °C. The aqueous phase, heated at 75 °C, was then dropped into the lipid matrix, under continuous stirring. Then, the pre-emulsion was immediately cooled and sonicated for 15 min. Finally, formulations were stored at 4 °C and analyzed 24 h after preparation.

### Nanoparticle characterization

Mean particle size (Z-Ave), polydispersity index (PDI), Zeta potential (ZP), physical appearance, pH value and physical stability were determined for unloaded (NLC0) and Myr-NLC (NLC1, NLC2) formulations. The mean size and PDI were determined by photocorrelation spectroscopy (dynamic light scattering) with the instrument NanoSizer ZS90 (Malvern Instruments, UK). Samples were ten-fold diluted with distilled water before mean size and PDI analyses. Values are reported as mean ± SD from 90 measurements (three sets of 10 measurements in triplicate). The ZP values were calculated with the software of NanoSizer ZS90 (Malvern Instruments, UK); using the Smoluchowski equation and including the average values of electrophoretic nanoparticle mobility. ZP values are reported as the mean of three sets of 100 measurements. The pH values were measured with a pH-meter XS INSTRUMENTS mod pH 510 (Carpi Monza - Italy).

### Nanoparticle stability studies

Stability of NLC formulations upon storage was evaluated. NLC were stored in closed glass vials at 4 °C; size and physical appearance were evaluated 1, 3, and 6 months after NLC preparation. Data of stored NLC were recorded and compared with NLC preparation at time zero.

### Formulation sterilization

NLC sterilization was carried out with several sterilization filters. Evaluation of NLC physical characteristics was carried out and compared to pre-sterilized NLC. To choose the most suitable filter, sterilization steps were carried out at first on unloaded NLC with different syringe filters (0.22 µm membrane mesh): (i) 4-mm disposable filter device with 0.2 µm nylon (NYL) membrane (Polypropylene housing) (Whatman); (ii) 25-mm disposable filter device with 0.2 µm polyethersulfone (PES) membrane (GD/X) (Whatman); (iii) 13-mm disposable filter with 0.2 µm polytetrafluorethylene (PTFE, Hydrophilic Fluoropore®) membrane (Millex^®^-LG). Nanoparticle size was evaluated immediately after NLC preparation, then NLC formulations were sterilized and re-analyzed, to evaluate preservation of characteristic pre-filtered NLC properties.

### Myriocin extraction procedure from biological samples

Myriocin was extracted as described by Campisi et al. with some modifications (Campisi et al., 2017). Weighted retinal samples were pretreated with 100 µl of PBS + 0.1% protease inhibitor, samples were then homogenized in the TissueLyser (Qiagen, Hilden, Germany) for 3 min at 50 oscillations/s. Rabbit retina homogenate (about 50 mg) was aliquoted, 70 µl and 30 µl for extraction of myriocin and sphingolipids, respectively. The homogenate of the mouse retina (about 5 mg) was used entirely for myriocin determination. The internal standard (50 µL, 14-OH myriocin 0.1 µg/ml) and 900 µl of distilled water were added to the tissue homogenate, then sonicated for 30 min. After centrifugation for 5 min at 10000 rpm, the supernatant was loaded on Strata X 30 mg cartridges (Phenomenex, CA, USA) connected to Supelco Visiprep Dil solid phase extraction (SPE) machine. Cartridges were previously conditioned with methanol (MeOH, 1 ml) and distilled water (1 ml). The cartridges were rinsed with 15% MeOH (1 ml), and then with 50% MeOH. Cartridges were dried under vacuum for 5 min, to remove excess water. The retained extracted sample was then eluted with MeOH/acetonitrile (1:1) + 0.1% formic acid (1 ml). The eluate was subjected to a gentle nitrogen stream, until dryness. Finally, the product of SPE purification was re-dissolved into 150 µl acetonitrile/water (1:1). Then, 10 µL of the sample were injected into LC-MS/MS instrumentation for quantitative analysis. Rabbit’s vitreous was filtered through a nylon filtering membrane (NY, mesh diameter 0.45 µm). The internal standard was added to purified samples (50 µL, 14-OH myriocin 0.1 µg/mL), then 10 µL of sample were directly injected into the LC-MS/MS.

### Sphingolipid extraction procedure from biological samples

The sphingolipid extraction was modified compared to the protocol previously published by Dalmau et al. (2015). Rabbit retinal homogenate (30 µL) was diluted with 70 µL of PBS + 0.1% protease inhibitor. Proteins were quantified with the Bradford method. The internal standard was added to the purified sample (10 µl, Cer C12 20 mM) along with a mixture of MeOH/chloroform (2:1, v/v), then sonicated for 30 min. Samples were then incubated overnight in an oscillator bath at 48 °C. Once at room temperature, 75 μl of 1M KOH in MeOH were added to the samples, then subjected to 2 h incubation at 37 °C. The pH was adjusted at 7.0 by adding 75 μl 1M acetic acid in MeOH. After evaporation with a gentle stream of nitrogen, samples were dissolved in 150 μl of MeOH and then centrifuged for 10 min at 13000 rpm. After this, 10 µL of purified samples were directly injected into the LC-MS/MS instrument for quantitative analysis.

### LC-MS/MS instrument and analysis parameters

The analytical system consisted of a UPLC Dionex 3000 UltiMate (Thermo Fisher Scientific, USA) connected to an ABSciex 3200 QTRAP with electrospray ionization TurboIonSpray™ source (AB Sciex S.r.l., Milano, Italy). The LC-MS/MS analysis of myriocin was carried out according to Campisi et al. (2017), while the sphingolipids analysis was carried out as described by Merrill et al. (2005). Technical details are thoroughly explained in the Supplementary material.

### Analytical performance of myriocin and sphingolipids analysis

For myriocin analysis, the calibration curves were built by adding up to 200 ng/vial to the blank matrix. Linearity was observed in the whole range of the calibration curve (*y = 0.8185x − 0.0455, R^2^* *= 0.9991*). Precision and accuracy of the myriocin analytical method were previously evaluated (Campisi et al., 2017) and confirmed in the present study. The limit of quantification (LOQ) was 0.1 ng/vial, as calculated by Multiquant software 2.1. The accuracy of the method was between 80–120% and the CV%<20%. The limit of detection (LOD) was 0.05 ng/vial, corresponding to a signal-to-noise ratio greater than 3, according to the ICH Validation of Analytical Procedures text and methodology. For sphingolipids analysis, six-point calibration curves were built by adding increasing amounts of a standard mixture of 12 ceramide species in water in the concentration range 0–40 pmol/vial; the linearity was observed for each ceramide (R^2^>0.99).

### Ocular pharmacokinetics and tolerability

Our experimental protocol complied with the statements of the Association for Research in Vision and Ophthalmology (ARVO) for the use of animals in ophthalmic and visual research. New Zealand (NZ) albino rabbits (weight 2.0–2.5 kg) were purchased from Envigo s.r.l (San Piero al Natisone, Udine, Italy). Rabbit can be considered a reliable animal model for translational ocular pharmacokinetics studies (Durairaj, 2017). Rabbits were housed in standard conditions for one week before the experiment (light-controlled room, controlled temperature 22 ± 1 °C and humidity 10–30%). Rabbits were randomly assigned to two experimental groups (treated and vehicle): *n* = 10 per experimental group, 2 rabbits and 4 retinas per time point. The two experimental groups of rabbits received ocular topical administration, 30 µl × 2 times within 5 min of the interval, of Myr-NLC (NLC1, drug loading 0.68 mg/ml) and vehicle (unloaded NLC), respectively. Rabbits were sacrificed at 30, 60, 120, 180, 240 min after ocular topical administration of the myriocin-NLC and unloaded NLC. Rabbits were sacrificed by intravenous administration of 0.3 ml/kg of Tanax^©^ (Intervet, Milano, Italy), after sedation with an intramuscular administration of 10 mg/kg of Zoletil^©^ (Virbac, Milano, Italy). Eyes were enucleated and ocular tissues (vitreous and retina) were collected. Tissue samples were stored at −80 C° until quantitative analysis of myriocin. Ocular distribution of myriocin was determined also in the retina of C57BL6J mice after topical administration of myriocin-NLC formulation (NLC1, drug loading 0.68 mg/ml). In order to increase formulation corneal residency, three 5 µl administrations within 2 min of interval were carried out. The amount of topical administered myriocin was 15 µg. C57BL6J mice (*n* = 4) per group were sacrificed 30, 60, 180 and 240 min after ocular topical administration of myriocin-NLC formulation; a separate set of animals (*n* = 12) was used to determine myriocin in control mice (vehicle). Mice were sacrificed by cervical dislocation; the eye enucleated and retina collected and stored at −80 °C until analysis.

The following pharmacokinetics parameters were determined in rabbit and mouse eye tissues: peak eye tissue concentration (*C_max_*), and time of peak of eye tissue concentration (*T_max_*). The area under the curve (*AUC*_0-240_) of eye tissues was extrapolated from the concentration of myriocin [myriocin] *vs.* time curves. Ocular safety of unloaded NLC and Myr-NLC formulations were evaluated by a modified Draize’s test in a separate set of rabbits (*n* = 6) (Leonardi et al., 2014; Giannavola et al., 2003). Formulations were topically administered (30 μl) in the eye every 30 min for 6 h (12 treatments). Scores were assigned accordingly to previous studies, and eyes were examined at 10 min and 6 h after Myr-NLC administration by a slit lamp (4179 T Sbisà, Florence, Italy). Observations were made by two independent observers in a masked way.

### Statistical analysis

The software GraphPad Prism (version 5.0; GraphPad Software, San Diego, CA, United States) was used to carry out statistical analysis and graph design. All data are expressed as mean values ± standard deviation (SD). The results were analyzed using one-way ANOVA. Two-way ANOVA was used for comparison of [myriocin] *vs.* time curves. Tukey–Kramer *post-hoc* multiple comparisons test was also carried out. Differences between groups were considered significant given *p*-values < .05.

## Results

### Preparation and characterization of the NLC system

NLC were prepared accordingly to an adapted melt-emulsification and ultrasonication technique. Lipid matrix and aqueous phase’s composition were chosen on the basis of different experimental tests (data not shown). Chemical content of formulations is shown in Table 1.

**Table 1.** Composition (%, w/v) of unloaded NLC and Myr-NLC formulations.

	Gelucire 44/14	Mygliol 812	Tween 80	Benzalkonium chloride	Myr theoretical concentration (mg/ml)	Drug loading (mg/ml)*	% Encapsulation efficiency
NLC 0	10	5	2.5	0.05	–	–	–
NLC 1	10	5	2.5	0.05	1	0.68 ± 0.02	68.0
NLC 2	10	5	2.5	0.05	2	1.60 ± 0.003	80.0

*Drug loading was determined in three separate samples by means of HPLC-MS/MS.

Mean particle size (Z-Ave), polydispersity index (PDI), Zeta potential (ZP), physical appearance, pH value and physical stability were determined for unloaded and myriocin-NLC formulations (Table 2).

**Table 2.** Characteristics of NLC formulations.

Batch	Z-Ave (nm)	PDI	ZP (mV)	Physical appearance	pH
NLC 0	86.25 ± 1.530	0.395 ± 0.006	3.81 ± 0.814	Clear	5.73
NLC 1	91.33 ± 3.153	0.658 ± 0.05	1.06 ± 0.179	Lightly milky	5.01
NLC 2	126.7±3.595	0.496 ± 0.002	2.48 ± 0.188	Milky	5.10

### Stability studies

Due to the limited stability of myriocin at temperatures above 0 °C, NLC stability was evaluated upon storage in closed glass vials at 4 °C. Mean particle size, PDI, pH and physical appearance were evaluated for up to 6 months after preparation. We found that both unloaded and Myr- NLC were physically stable for the tested period of storage (Table 3). The NLC2 formulation showed a tendency to increase the mean particle size starting from the third month of storage, however maintaining the mean particle size below 200 nm, which is suitable for topical ophthalmic administration.

**Table 3.** Stability of NLC and Myr-NLC formulations.

	Z-Ave (nm)	PDI	pH	App
*Time 0*				
NLC0	86.25 ± 1.530	0.395 ± 0.006	5.73	Clear
NLC1	91.33 ± 3.153	0.658 ± 0.050	5.01	Lightly milky
NLC2	126.70±3.595	0.496 ± 0.002	5.10	Milky
*1 month*				
NLC0	93.58 ± 0.351	0.316 ± 0.034	5.56	Clear
NLC1	116.51 ± 0.833	0.273 ± 0.007	5.06	Lightly milky
NLC2	134.80 ± 2.06	0.380 ± 0.013	5.09	Milky
*3 months*				
NLC0	102.31 ± 0.521	0.250 ± 0.001	5.55	Clear
NLC1	119.32 ± 1.570	0.244 ± 0.006	5.04	Lightly milky
NLC2	166.22 ± 5.781	0.474 ± 0.074	5.12	Milky
*6 months*				
NLC0	134.90 ± 0.794	0.216 ± 0.022	5.58	Lightly milky
NLC1	122.22 ± 0.195	0.188 ± 0.015	5.20	Lightly milky
NLC2	189.99 ± 4.222	0.313 ± 0.111	5.31	Milky

### Sterilization

Unloaded NLC formulations were sterilized after preparation; an aliquot of each sample was sterilized using three syringe sterile filters. Although, filtration of unloaded NLC0 was quite easy, the nylon and PTFE 0.2 µm membranes exerted a certain resistance to filtration of the Myr- NLC formulation. The PES sterile filter provided an easy filtration of the Myr-NLC formulation (NLC1), preserving the characteristics of the pre-filtered NLC batch (mean particle size was 91.33 and 82.34 nm, before and after filtration, respectively).

### Ocular distribution of the Myr-NLC formulation (NLC1) in NZ rabbits and C57BL6J mice

We carried out preliminary comparison of Myr-NLC retinal distribution with myriocin aqueous suspension (PBS pH 7.4, [myriocin] = 1 mg/ml) and a Myr-SLN formulation (see supplemental material). Myr-SLN formulation was prepared as previously reported, without addition of cationic lipid didecylmethylammonium bromide (Amadio et al., 2016; Leonardi et al., 2015) (Supplemental material).

NLC1 formulation provided significantly (*p* < .0001) higher myriocin retinal availability, after topical ocular administration, compared to myriocin suspension or Myr-SLN formulation (supplemental material).

We administered 40.8 µg of myriocin (two administration of 30 µl of NLC1, drug loading = 0.68 mg/ml) to rabbit eye by topical route, while mice received 10.2 µg myriocin (three administration of 5 µl of NLC1, drug loading = 0.68 mg/ml). We used two different species for pharmacokinetics study because rabbits are well known valuable animal model to assess ocular PK (Durairaj, 2017), while mice were previously used as genetic animal model of retinitis pigmentosa (Strettoi et al., 2010). Indeed, we evaluated myriocin retinal distribution in mice in order to confirm that Myr-NLC formulation is able to guarantee pharmacological levels of drug.

**Figure 1.**
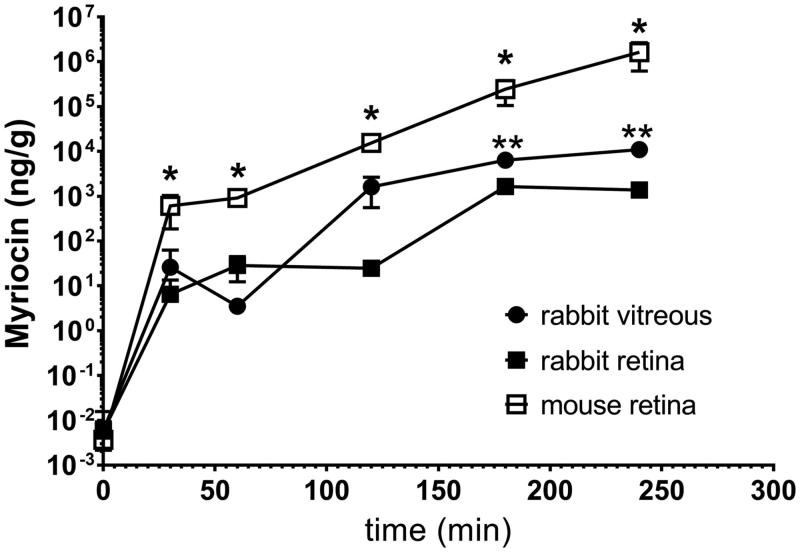
Myriocin (topically administered as a myriocin-NLC) distribution in rabbit vitreous and retina. *p < .05 mouse retina *vs.* rabbit vitreous and retina. **p < .05 rabbit vitreous *vs.* rabbit retina.

Figure 1 shows myriocin distribution in rabbit vitreous and retina after topical administration of Myr-NLC (NLC1) (Table 4). Myriocin levels in rabbit retina were significantly lower (*p* < .05) compared to rabbit vitreous levels, 180 and 240 min after ocular topical administration of the Myr-NLC formulation (Figure 1).

**Table 4.** Ocular PK parameters of the myriocin-NLC (NLC1) formulation.

Tissue	T_max_ (minutes)	C_max_ (ng/g)	AUC_0-204_ (ng*min /g)
Mouse retina	240	2030061 ± 871475	80556900 ± 9611659*†
Rabbit vitreous	240	10996 ± 745	810507 ± 80068*
Rabbit retina	180	1646 ± 240	142789 ± 14898*

*p < .05 AUC_0-240_ rabbit vitreous *vs*. rabbit retina; †*p* < .001 mouse retina *vs*. rabbit vitreous/rabbit retina.

Furthermore, Figure 1 shows myriocin distribution in the retina of Myr-NLC (NLC1) treated mice (Table 4). The myriocin availability in mouse retina was significantly (*p* < .05) higher than in rabbits (vitreous and retina; Figure 1 and Table 4). Myriocin levels detected in mouse retina at 120 min (2.2 × 10^4^ ± 0.5 × 10^4^ ng/g), 180 min (3.0 × 10^5^ ± 0.8 × 10^5^ ng/g) and 240 min (2.0 × 10^6^± 0.9 × 10^6^ ng/g) were significantly (*p*<.05) higher than retinal drug levels at 30 min and 60 min.

Pharmacokinetic parameters (*T_max_*, *C_max_* and *AUC*_0 − 240_) were extrapolated from [myriocin] *vs.* time curves (Figure 1) and reported in Table 4.

Pharmacokinetics parameters of myriocin distribution in rabbit vitreous are expressed as ng/g (*C_max_* values) or ng*min/g (*AUC*), assuming vitreous density equal to 1 (Struble et al. 2014). Furthermore, we found that unloaded NLC and Myr-NLC (NLC1) were well tolerated by rabbits. The scores of the modified Draize’s test were 0, at 10 min and 6 h (data not shown).

### Inhibition of *de novo* sphingolipids biosynthesis by myriocin

**Figure 2.**
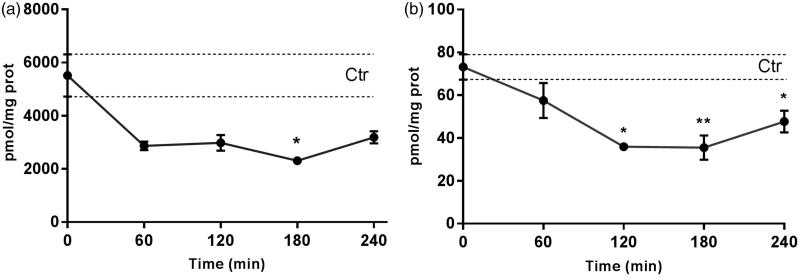
Ceramides (A) and dihydroceramides (B) levels in the retina after topical administration of the myriocin-NLC. Ceramide or dihydroceramide range in control animals is represented by two dotted lines. *p < .05 **p < .001 Myr-NLC rabbits *vs.* vehicle-treated group.

Total ceramides and dihydroceramides levels in the rabbit retina were determined to evaluate the efficacy of the myriocin delivered with NLC to the back of the eye (Figure 2). Myr-NLC treatment decreased ceramides and dihydroceramides levels in the rabbit retina, compared to control rabbits (vehicle). Retinal ceramide levels were found to be significantly (*p* < .05) decreased in the Myr-NLC-treated group compared with the vehicle-treated group, 2312 ± 150 pmol/mg*prot and 5518.5 ± 794.9 pmol/mg*prot, respectively (180 min after treatment). Dihydroceramides levels were found to be significantly (*p* < .001) decreased in the rabbit retina 120 min (35.9 ± 1.8 pmol/mg*prot), 180 min (35.5 ± 5.7 pmol/mg*prot) and 240 min (47.7 ± 5.1 pmol/mg*prot) after treatment with Myr-NLC, compared to control rabbits (73.2 ± 6 pmol/mg*prot).

## Discussion

The eye is characterized by several barriers that limit the drug bioavailability after topical ocular administration. In general, only 1–10% of topical administered drugs is absorbed and about 1% of any instilled drug reaches the aqueous humor (Bucolo et al., 2012; Macha et al., 2003). Therefore, drug delivery targeting the retina is a challenging task, generally by-passed with intravitreal injections. Currently, anti-VEGF drugs used in clinical practice to treat age-related macular degeneration are injected intravitreally. Recently, intravitreal injection of myriocin was also explored in mice to assess retinal bioavailability by this route (Campisi et al., 2017). However, intravitreal injections may damage eye structures leading to retinal detachment, cataract, hyperemia, and endophthalmitis.

Intravitreal treatment with myriocin was also investigated on rd10 mice modeling RP demonstrating a robust reduction of ceramide retinal levels (Strettoi et al., 2010). The topical ophthalmic approach was successfully explored (Strettoi et al., 2010) using solid lipid nanoparticle (SLN) formulation of myriocin in rd10 mice. Significant rescue effects on photoreceptor survival and retinal function were found, reflected in the preservation of the a-wave of ERG in rd10 mice treated with myriocin (Piano et al., 2013).

Exploitation of nanotechnological approaches led to successful drug delivery to the back of the eye in pre-clinical studies (Altamirano-Vallejo et al., 2018; Papangkorn et al., 2018; Mahaling et al., 2018; Bucolo et al., 2011); a fruitful approach, among others, is represented by solid lipid nanocarriers (SLN) (Battaglia et al., 2016; Leonardi et al., 2015; Chetoni et al., 2016). In the present study, we exploited the use of nanostructured lipid carriers (NLC) that represents a technological evolution of SLN, to deliver myriocin. Main advantages of NLC are related to a higher physical stability and drug loading capacity, compared to SLN. NLCs are obtained by mixing two different lipids, one solid and the other liquid, at room temperature. This protocol leads to the formation of a hydrophobic core in the NLC, with structural in-homogeneities, where drugs can be easily dissolved and encapsulated in tiny oil compartments. These chemical-physical properties characterize the high loading capacity and negligible drug expulsion of NLC (Müller et al., 2002).

We demonstrated that the Myr-NLC formulation was characterized by high drug loading capacity. Furthermore, the formulation retains considerable stability at 6 months from formulation preparation. Myr-NLC formulation stability was evaluated at 4 °C, because myriocin is not stable at range temperature generally used for formulation stability assessment, as reported by International Council for Harmonization (ICH) guidelines. However, our next goal is to follow up this preliminary study with a pre-industrial optimization of the formulation, including (ICH) accelerated stability tests. Both Myr-NLC and unloaded NLC eye drops were well tolerated by the rabbit eye. We also characterized, for the first time, the ocular distribution of the topical ocular Myr-NLC formulation in rabbits and mice. The Myr-NLC formulation efficiently delivered myriocin to the back of the eye of both treated mice and rabbits, after single topical ocular administration. We hypothesize that the Myr-NLC formulation provides a more robust drug bioavailability to the back of the eye through preferential conjunctival/scleral drug absorption, with respect to the trans-corneal route. Moreover, the myriocin-NLC worked as a depot formulation, due to its long corneal residence time, because myriocin (Myr-NLC) reached the maximum concentration in the back of the eye of rabbits and mice after 240 min, in agreement with previous studies (Araujo et al., 2012; Schopf et al., 2015). The average weight of rabbit vitreous is one gram (Struble et al., 2014); therefore, rabbit vitreous levels of myriocin were ∼10 µg (vitreous C_max_* 1 g), after administration of the myriocin-NLC (1 mg/ml) formulation. Moreover, the amount of myriocin that reached the mouse retina was 5.8 µg (*C_max_*) after Myr-NLC topical ocular treatment. Therefore, myriocin levels both in rabbit vitreous and mouse retina are in the therapeutic range previously determined in the rd10 mouse model of RP (Strettoi et al., 2010). In the present study we demonstrated that Myr-NLC formulation was able to provide pharmacological drug levels in rabbit retina. Considering that myriocin acts as sphingolipid synthesis inhibitor, the remarkable drug levels in rabbit retina are able to decrease ceramides and dihydroceramides, as previously demonstrated (Strettoi et al., 2010). Therefore, our data suggest a potential therapeutic effect of Myr-NLC to manage retinitis pigmentosa.

## Conclusion

Our results demonstrated that the Myr-NLC formulation is able to deliver effective levels of myriocin to the back of the eye both in rabbits and mice. Myriocin levels, delivered to rabbit vitreous and mouse retina, overlapped the pharmacological effective doses as previously demonstrated *in vivo* (Strettoi et al., 2010). The efficacy of Myr-NLC treatment was also proven by the significant decrease of ceramides and dihydroceramides levels in Myr-NLC rabbit retina, compared to vehicle-treated group. In conclusion, these data suggest that the Myr-NLC ophthalmic formulation is suitable for pharmaceutical development and warrants further clinical evaluation for the treatment of retinitis pigmentosa.

## Supplementary Material

SUPPL_MATERIAL_r1.docx
