# Supplementary material for: Novel ophthalmic formulation of myriocin: implications in retinitis pigmentosa
Source: Drug Deliv. 2019 Mar 11;26(1):237–43. doi: 10.1080/10717544.2019.1574936 (PMC6419690; doi:10.1080/10717544.2019.1574936)
Supplement: SUPPL_MATERIAL_r1.docx [file IDRD_A_1574936_SM3847.docx]

**SUPPLEMENTARY MATERIALS**

*1. LC-MS/MS conditions for myriocin analysis:*

The analytical system consisted of a HPLC coupled to a tandem mass spectrometer. The liquid chromatograph system was a Dionex 3000 UltiMate instrument with autosampler, binary pump and column oven (Thermo Fisher Scientific, USA). Separation was carried out on a reversed-phase Inertsil ODS3 column, 150 × 3.0 mm, 3 μm particle size (GL Sciences, Tokyo, Japan) analytical column. The analytical column was preceded by a security guard cartridge. We applied a linear gradient by mixing eluent A (water + 0.1% formic acid) and eluent B (acetonitrile). The column was equilibrated with 50% of B, and B was increased to 100% in 5 min. This condition was held for 0.5 min, therefore the initial condition (50% of B) was reached in 0.5 min and kept for 2 min. The flow rate was 0.4 mL/min, the autosampler and the column oven were kept at 15°C and 45°C, respectively. Retention times of myriocin and IS were 3.6 and 3.3, respectively.

The tandem mass spectrometer was an AB Sciex 3200 QTRAP instrument with electrospray ionization TurboIonSpray™ source (AB Sciex, Milan, Italy). Instruments were managed with the proprietary manufacturer's software and according to the manufacturer's instructions. The analytical data were processed using Analyst software (version 1.2). The ion spray voltage was set at 3.5 kV and the source temperature was set at 600 °C. Nitrogen was used as a nebulizing gas (GS 1, 45 psi), turbo spray gas (GS 2, 55 psi) and curtain gas (30 psi). The collision-activated dissociation (CAD) was set to a medium level. The dwell time was set at 0.1 s, and the MS scan was performed in negative ion modes (ESI-). MS/MS experiments were carried out using nitrogen as collision gas. Compound-dependent parameters were optimized *via* direct infusion. Multiple reaction monitoring (MRM) mode was used. Quantitative analysis was performed interpolating the area ratio (peak area of quantifier ion of myriocin/area IS) with calibration curve. When necessary, quantification based on absolute myriocin area was performed in order to avoid possible metabolite interferences. Linearity was confirmed also using the quantification procedure based on absolute area.

**Table S1**. MS conditions for each analyte, in bold transition used for quantification

| **Analytes** | **Transition** | **DP (eV)** | **EP (V)** | **CE (V)** |
| --- | --- | --- | --- | --- |
| Myriocin | **400.3>104.0** | **-50** | **-5** | **-28** |
|  | 400.3>334.3 | -50 | -5 | -24 |
| 14-OH Myriocin (IS) | 402.3>104.0 | -45 | -5 | -24 |
|  | **404.3>336.0** | **-45** | **-5** | **-24** |

*2. LC-MS/MS conditions for sphingolipids analysis:*

The analytical system consisted of a HPLC coupled to a tandem mass spectrometer. The liquid chromatograph system was a Dionex 3000 UltiMate instrument with autosampler, binary pump and column oven (Thermo Fisher Scientific, USA). Separation was attained on a reversed-phase BEH C-18 10 x2.1 x1.7 μm analytical column preceded by a security guard cartridge. Linear gradient was obtained by mixing eluent A (water + 2 mM ammonium formate + 0.2% formic acid) and eluent B (methanol + 1 mM ammonium formate + 0.2% formic acid). The column was equilibrated with 80% of B, therefore B was increased to 90% in 3 min, held for 3 min, increased to 99% (B) in 9 min, held for 3 min. After that, the initial condition (80% of B) was obtained in 2 min and kept for 2 min. The flow rate was 0.3 mL/min, the autosampler and the column oven were kept at 15°C and 30°C, respectively.

The tandem mass spectrometer was an AB Sciex 3200 QTRAP instrument with electrospray ionization TurboIonSpray™ source (AB Sciex, Milano, Italy). Instruments were managed with the proprietary manufacturer's software and according to the manufacturer's instructions. The analytical data were processed using Analyst software (version 1.2). The ion spray voltage was set at 5.5 kV and the source temperature was set at 300 °C. Nitrogen was used as a nebulizing gas (GS 1, 45 psi), turbo spray gas (GS 2, 50 psi) and curtain gas (25 psi). The collision-activated dissociation (CAD) was set to a low level. The dwell time was set at 0.1 s, and the MS scan was performed in positive ion modes (ESI+). MS/MS experiments were conducted using nitrogen as collision gas. Compound-dependent parameters were optimized *via* direct infusion. Multiple reaction monitoring (MRM) mode was used. Quantitative analysis was performed interpolating each peak area of analyte/area IS with calibration curve of each sphingolipids. The sphingolipids amount was normalized by total protein content, expressed in milligram, in each samples.

**Table S2.** MS conditions and retention times for each sphingolipids

| **Analytes** | **Transition** | **R_T_** | **DP (eV)** | **CE (V)** |
| --- | --- | --- | --- | --- |
| Cer C12 (IS) | 482.7 > 264.4 | 7.32 | 40 | 29.0 |
| Cer C14 | 510.7 > 264.4 | 8.41 | 40 | 29.5 |
| Cer C16 | 538.8 > 264.4 | 9.78 | 40 | 32.5 |
| Cer C18 | 566.8 > 264.4 | 11.19 | 40 | 34.5 |
| Cer C18:1 | 564.8 > 264.4 | 10.27 | 40 | 35.5 |
| Cer C20 | 594.8 > 264.4 | 12.52 | 40 | 36.0 |
| Cer C22 | 622.9 > 264.4 | 13.73 | 40 | 37.5 |
| Cer C24 | 650.9 > 264.4 | 14.84 | 40 | 41.5 |
| Cer C24:1 | 648.9 > 264.4 | 13.84 | 40 | 38.5 |
| DHCer C16 | 540.4 > 266.4 | 10.20 | 40 | 33.0 |
| DHCer C18 | 568.5 > 266.4 | 11.58 | 40 | 35.0 |
| DHCer C18:1 | 566.5 > 266.4 | 10.69 | 40 | 35.0 |
| DHCer C24 | 652.5 > 266.4 | 15.11 | 40 | 40.0 |
| DHCer C24:1 | 650.5 > 266.4 | 14.16 | 40 | 38.5 |

*4. NLC1 morphology*

Transmission electron microscopy (TEM) was performed using a Philips EM 400T microscope, (Eindhoven, Netherlands). Samples were prepared by deposition of a drop of diluted (100 folds) NLC1 suspension on the surface of a 200 mesh Formvar R -coated copper grid (TAAB Laboratories Equipment, Ltd., Aldermaston, UK), followed by a slow evaporation of water overnight at room temperature.

Electron microscopy showed a particle size compatible with the one estimated by photon correlation spectroscopy data of NLC1, reported in table 2 of main document (Figure 1S).

*Fig 1S. NLC1 nanoparticle TEM image.*

*
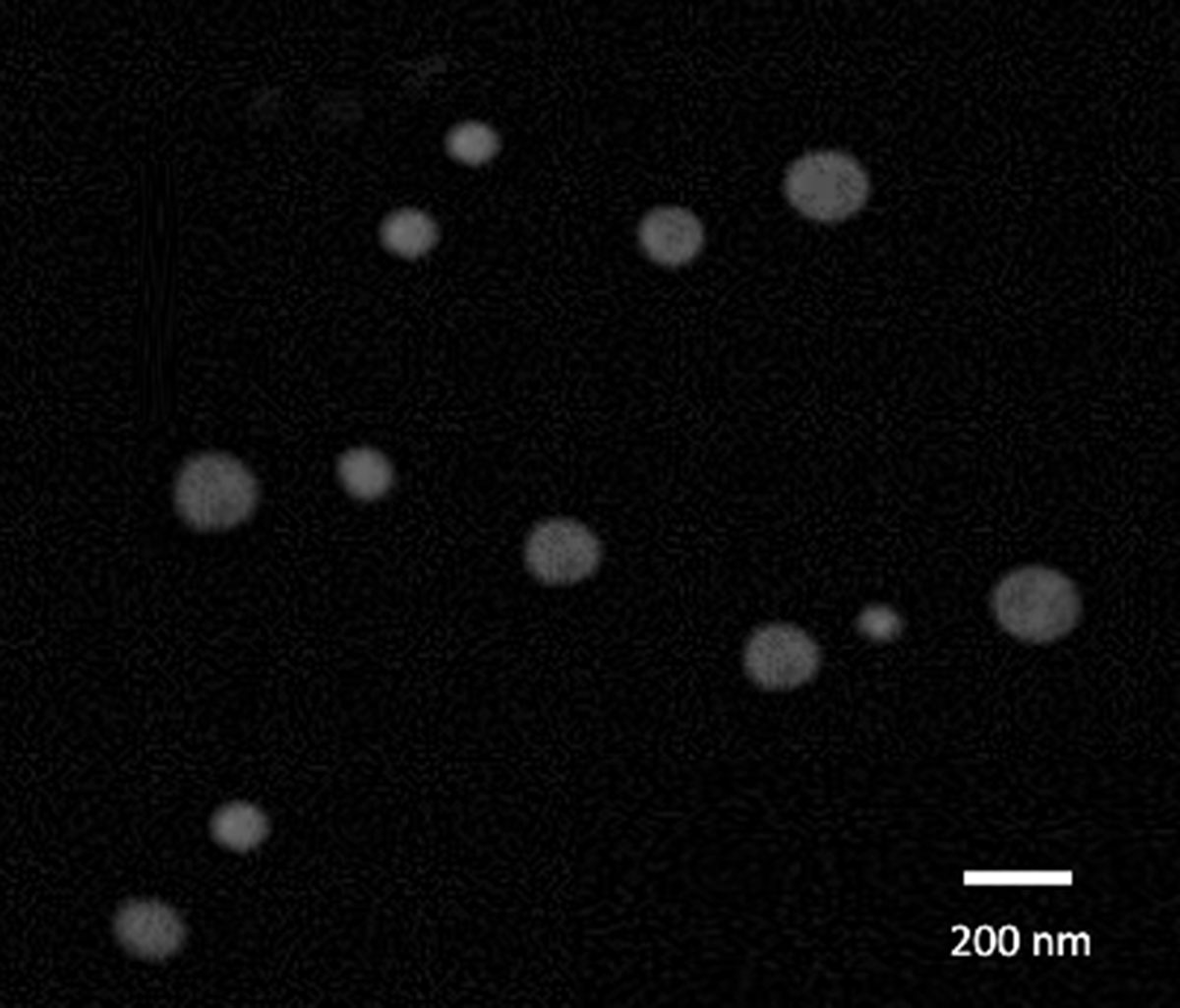
*

*4. PK profile of myriocin ophthalmic formulations.*

In a preliminary study, N=4 rabbits per each experimental group (1 rabbit, 2 retinas per time-time point) were treated with myriocin suspension, Myr-SLN and NLC1. Myr-SLN were prepared as described in (Amadio M. et al 2016 Pharmacological Research – Leonardi A. et al 2015 International Journal of Pharmaceutics), without addition of the cationic lipid didecylmethylammonium bromide. In particular, the lipid Softisan 100 was used at 1% w/v concentration and dissolved in acetone: ethanol 1:1 solution, which constituted the lipid phase. Myr-SLN beard a Z-ave = 221.6 ±1.2 and a PDI = 0.16 ± 0.02.

Rabbits were topically ocular treated with 60 µg of myriocin formulated as aqueous suspension (1 mg/ml of myriocin suspended in phosphate buffered saline, PBS pH 7.4). Other two groups of rabbits were treated with Myr-SLN (drug loading 0.55±0.07) and with NLC1 (drug loading 0.68±0.15). Myriocin retinal content has been determined as described in the methods section of main manuscript. The C_max_, T_max_ and AUC_0-240_ for retinal distribution of myriocin were determined and are listed in table S3.

As reported in table S3, myriocin retinal availability is higher of about 3-4 logs when delivered in nanostructured lipid carriers (Myr-NLC), in comparison to Myr-SLN and myriocin aqueous suspension. Therefore, the other pharmacokinetics in-vivo studies were carried out on rabbits and mice with the Myr-NLC formulation, as described in the main manuscripts.

Table S3. Comparison of pharmacokinetics parameters for retinal myriocin distribution, after topical administration of different myriocin ophthalmic formulation.

| Formulation | C_max_ (ng/g) | T_max_ (min) | AUC_0-240_ (ng*min /g) |
| --- | --- | --- | --- |
| Myriocin suspension  (1 mg/ml) | 1.55±0.50 | 60 | 169.4±26.7 |
| Myr-SLN  (drug loading 0.55 mg/ml) | 0.55±0.25 | 240 | 66.2±15.5 |
| Myr-NLC (NLC1, drug loading 0.68 mg/ml) | 1451±370 | 180 | 185192±36000 |
